# Supplementary material for: Co-Assembly of 40S and 60S Ribosomal Proteins in Early Steps of Eukaryotic Ribosome Assembly
Source: Int J Mol Sci. 2019 Jun 8;20(11):2806. doi: 10.3390/ijms20112806 (PMC6600400; doi:10.3390/ijms20112806)
Supplement: Supplementary file 1 [file ijms-20-02806-s001.pdf]

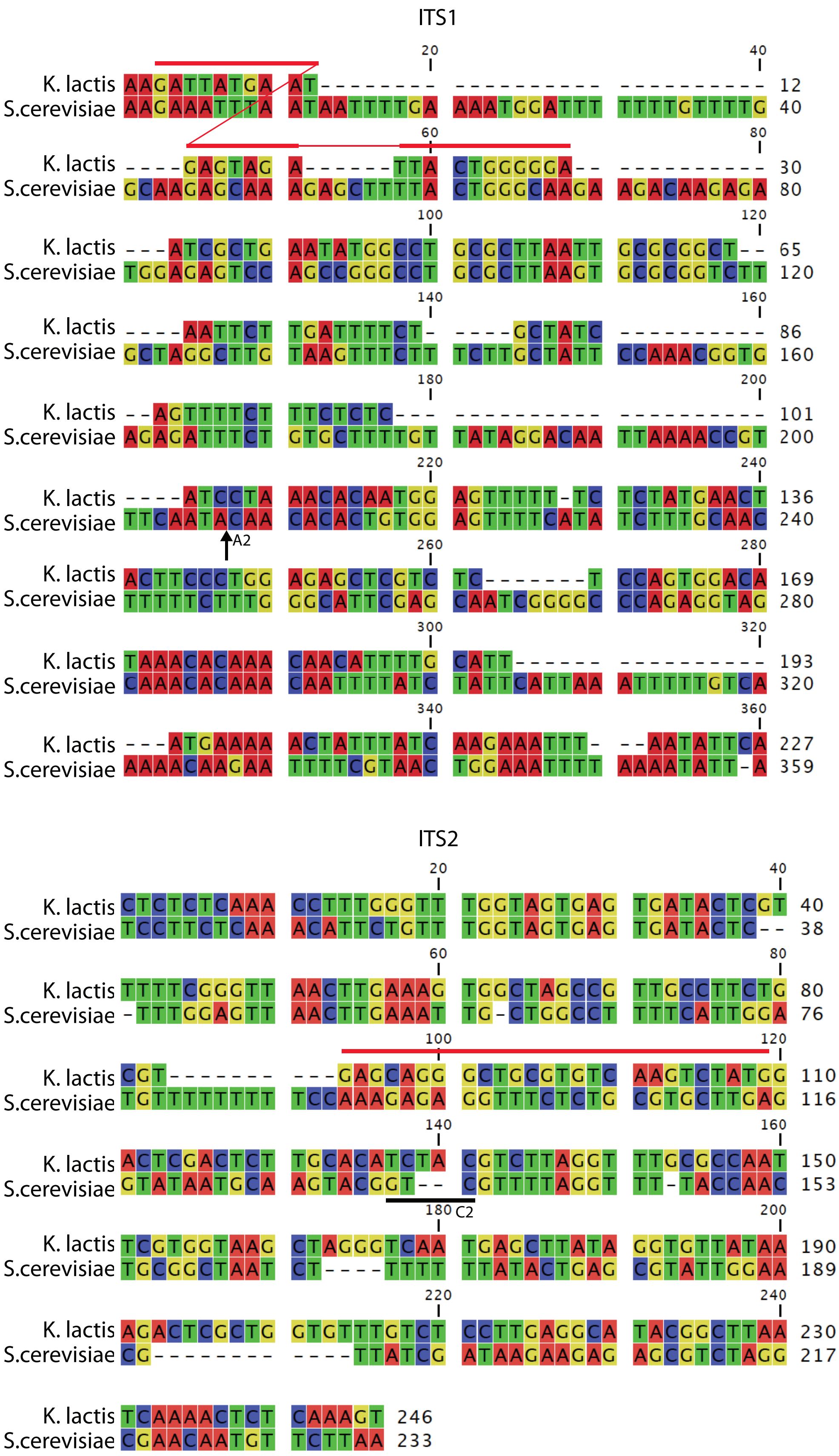
ITS1
K. lactis AAGATTATGA AT-------- ---------- ---------- 12
S.cerevisiae AAGAAATTTA ATAATTTTGA AAATGGATTT TTTTGTTTTG 40
K. lactis ----GAGTAG A------TTA CTGGGGGA-- ---------- 30
S.cerevisiae GCAAGAGCAA AGAGCTTTTA CTGGGCAAGA AGACAAGAGA 80
K. lactis ---ATCGCTG AATATGGCCT GCGCTTAATT GCGCGGCT-- 65
S.cerevisiae TGGAGAGTCC AGCCGGGCCT GCGCTTAAGT GCGCGGTCTT 120
K. lactis ----AATTCT TGATTTTCT- ----GCTATC ---------- 86
S.cerevisiae GCTAGGCTTG TAAGTTTCTT TCTTGCTATT CCAAACGGTG 160
K. lactis --AGTTTTCT TTCTCTC--- ---------- ---------- 101
S.cerevisiae AGAGATTTCT GTGCTTTTGT TATAGGACAA TTAAAACCGT 200
K. lactis ----ATCCTA AACACAATGG AGTTTTT-TC TCTATGAACT 136
S.cerevisiae TTCAATACAA CACACTGTGG AGTTTTCATA TCTTTGCAAC 240
A2
K. lactis ACTTCCCTGG AGAGCTCGTC TC-------T CCAGTGGACA 169
S.cerevisiae TTTTTCTTTG GGCATTCGAG CAATCGGGGC CCAGAGGTAG 280
K. lactis TAAACACAAA CAACATTTTG CATT------ ---------- 193
S.cerevisiae CAAACACAAA CAATTTTATC TATTCATTAA ATTTTTGTCA 320
K. lactis ---ATGAAAA ACTATTTATC AAGAAATTT- --AATATTCA 227
S.cerevisiae AAAACAAGAA TTTTCGTAAC TGGAAATTTT AAAATATT-A 359
ITS2
K. lactis CTCTCTCAAA CCTTTGGGTT TGGTAGTGAG TGATACTCGT 40
S.cerevisiae TCCTTCTCAA ACATTCTGTT TGGTAGTGAG TGATACTC-- 38
K. lactis TTTTCGGGTT AACTTGAAAG TGGCTAGCCG TTGCCTTCTG 80
S.cerevisiae -TTTGGAGTT AACTTGAAAT TG-CTGGCCT TTTCATTGGA 76
K. lactis CGT------- ---GAGCAGG GCTGCGTGTC AAGTCTATGG 110
S.cerevisiae TGTTTTTTTT TCCAAAGAGA GGTTTCTCTG CGTGCTTGAG 116
K. lactis ACTCGACTCT TGCACATCTA CGTCTTAGGT TTGCGCCAAT 150
S.cerevisiae GTATAATGCA AGTACGGT-- CGTTTTAGGT TT-TACCAAC 153
C2
K. lactis TCGTGGTAAG CTAGGGTCAA TGAGCTTATA GGTGTTATAA 190
S.cerevisiae TGCGGCTAAT CT----TTTT TTATACTGAG CGTATTGGAA 189
K. lactis AGACTCGCTG GTGTTTGTCT CCTTGAGGCA TACGGCTTAA 230
S.cerevisiae CG-------- ----TTATCG ATAAGAAGAG AGCGTCTAGG 217
K. lactis TCAAAACTCT CAAAGT 246
S.cerevisiae CGAACAATGT TCTTAA 233

Supplementary Figure 1. Design of hybridization probes for Kluyveromyces lactis ITS1 and ITS2. The sequences of ITS1 and ITS2 in *K. lactis* was aligned with the corresponding spacers in *S. cerevisiae* using CLC Sequence Viewer 8. The arrow below the alignments indicate the 3' end points after cleavage at A2 and C2 in the *S. cerevisae* ITS1 and ITS2, respectively (van Nues et al. 1994; van Nues 1995). The red lines above the K. lactis sequences indicate the sequences that are complementary to the *K. lactis*-specific probes. Note that the *K. lactis* ITS1 probe spans two insertions in *S. cerevisiae* ITS1 that prevent hybridization of the *S. cerevisiae* ITS1 to the probe.

References for supplement

van Nues RW, Rientjes JM, van der Sande CA, Zerp SF, Sluiter C, Venema J, Planta RJ, Raue HA. 1994. Separate structural elements within internal transcribed spacer 1 of Saccharomyces cerevisiae precursor ribosomal RNA direct the formation of 17S and 26S rRNA. *Nucleic Acids Res* **22**: 912-919.

van Nues RW, Rientjes, J.M.J., Morrë, S.A., Mollee, E., Planta, R.J., Venema, J., Rauë,H.A. 1995. Evolutionarily conserved structural elements are critical for processing of internal transcibed spacer 2 from *Saccharomyces cerevisiae* precursor ribosomal RNA. *J Mol Biol* **250**: 24-36.
